# Molecular mechanism of 2′,3′-cGAMP degradation by monkeypox virus poxin-schlafen protein

**DOI:** 10.1016/j.jbc.2026.113163

**Published:** 2026-05-15

**Authors:** Yunxiao Huang, Benzhen Duan, Yang Xiao, Xiaoman An, Hongyu Zhao, Jingwen Wang, Fenglei Du, Baoyu Zhao

**Affiliations:** Shanghai Institute of Infectious Disease and Biosecurity, Key Laboratory of Medical Molecular Virology (MOE/NHC/CAMS), Shanghai Frontiers Science Center of Pathogenic Microorganisms and Infection, Department of Medical Microbiology and Parasitology, School of Basic Medical Sciences, Shanghai Medical College, Fudan University, Shanghai, China

**Keywords:** 2',3'-cGAMP, cGAS-STING pathway, innate immunity, monkeypox virus, poxin

## Abstract

The World Health Organization had declared the multiregional outbreak of monkeypox a global public health emergency twice since 2022. Poxin from orthopoxviruses degrades the second messenger 2′,3′-cGAMP of the cyclic GMP-AMP synthase-stimulator of interferon genes (cGAS-STING) pathway to evade innate immune surveillance. Monkeypox virus (MPXV) poxin is fused to schlafen and the mechanism of how it catalyzes the degradation of 2′,3′-cGAMP remains unclear. Here, we show that MPXV poxin-schlafen degrades 2′,3′-cGAMP into Gp[2′-5′]Ap[3′], suppressing interferon-β (IFN-β) induction mediated by the cGAS-STING pathway. The schlafen domain has no impact on the degradation of 2′,3′-cGAMP mediated by the poxin domain. Crystal structures of MPXV poxin domain and its complex with Gp[2′-5′]Ap[3′] reveal that 2′,3′-cGAMP binds to a pocket formed by two monomers of the poxin dimer and induces closure of the pocket to trigger degradation. MPXV poxin exhibits a strict substrate specificity and does not degrade 3′,3′-cGAMP, c-di-AMP, and c-di-GMP that also activate STING-mediated immune responses. These results unveil the molecular mechanism for 2′,3′-cGAMP degradation by MPXV poxin-schlafen protein and provide the structural basis for the development of inhibitors against MPXV infection.

Orthopoxvirus is a genus of dsDNA viruses in the Poxviridae family and includes several well-known pathogenic viruses such as variola virus (VARV), monkeypox virus (MPXV), vaccinia virus (VACV), cowpox virus (CPXV), and ectromelia virus (ECTV) (1, 2). Smallpox is a viral disease caused by variola virus and once a major cause of human mortality. The World Health Organization declared the global eradication of smallpox in 1980 (1). After the ending of smallpox vaccination, unvaccinated individuals were unable to obtain the cross protection provided by smallpox vaccine against other orthopoxviruses. Therefore, although smallpox had been eradicated through vaccination, the threat from orthopoxviruses persisted (2). In 2022, MPXV caused a global outbreak of monkeypox. Up to October 2025, more than 172,510 cases were confirmed in more than 140 countries and the World Health Organization declared monkeypox outbreak a public-health emergency of international concern (PHEIC) twice (https://www.who.int/health-topics/mpox) (https://worldhealthorg.shinyapps.io/mpx_global/_w_02641a24967341c6aab56c7b40761811/#overview). Based on phylogenetic analyses, MPXV can be divided into two major clades and four subclades: clade I (clade Ia and Ib) and clade II (clade IIa and IIb). Since 2022, the vast majority of monkeypox cases have been attributable to clade IIb (https://worldhealthorg.shinyapps.io/mpx_global/_w_02641a24967341c6aab56c7b40761811/#overview). MPXV infections usually cause some influenza-like symptoms, with fever, fatigue, lethargy, or myalgia, progression to pathognomonic skin lesions containing pustular papules, fluid-filled vesicles, and ulcerations (https://worldhealthorg.shinyapps.io/mpx_global/_w_02641a24967341c6aab56c7b40761811/#overview) (3).

The innate immune system is the first line of defense against invading pathogens (4). On sensing the pathogen-associated molecular patterns (PAMPs) such as DNA or RNA through pattern recognition receptors (PRRs), the innate immune system triggers the expression of a variety of cytokines including type-I interferon (IFN-I) to initiate host defense against viral or bacterial pathogens (5, 6). The cyclic GMP-AMP synthase (cGAS) is an important pattern recognition receptor that senses dsDNA in the cytosol (7, 8). On dsDNA binding, cGAS is activated and catalyzes the synthesis of a cyclic dinucleotide 2′,3′-cGAMP from ATP and GTP (9, 10, 11, 12). As a second messenger, 2′,3′-cGAMP binds and activates the adaptor protein stimulator of interferon genes (STING), which can further recruit and activate TANK-binding kinase 1 (TBK1) and IFN regulatory factor-3 (IRF-3) (7, 13, 14, 15). Activated IRF-3 dimerizes, translocates to the nucleus, and initiates IFN-β gene transcription (7, 16). As cGAS senses dsDNA in a sequence-independent manner, the cGAS-STING signaling pathway plays critical roles in immune responses against invading pathogens, autoimmune and inflammatory diseases, cancer, cardiovascular diseases, neurodevelopment, and so on (7, 17, 18, 19). Previous studies demonstrated that modified vaccinia virus Ankara (MVA) infection activated the cGAS-STING signaling pathway and elicited the production of IFN-I in murine conventional dendritic cells (cDCs) (20). ECTV infection induced the expression of IFN-I *via* the cGAS-STING pathway in murine cells (L929 and RAW264.7) (21). As expected, MPXV infection also stimulated IFN-I production through the activation of the cGAS-STING pathway. IFN-I treatment significantly reduced viral replication, alleviated splenomegaly in MPXV-infected CAST/EiJ mice and markedly reduced the severity of MPXV infection by alleviating skin lesions and lowering plasma viremia in rhesus macaques (22).

In order to productively replicate in host cells, orthopoxviruses need to evade innate immune surveillance. Indeed, many immunomodulatory proteins such as E5 and F17 were found in orthopoxviruses that inhibit the activation of the cGAS-STING signaling pathway (23, 24, 25). E5 mediated proteasome-dependent degradation of cGAS, while F17 dysregulated mTOR to simultaneously destabilize cGAS (24, 25). In addition, a nuclease called poxin was found in orthopoxviruses and could specifically degrade 2′,3′-cGAMP into linear Gp[2′-5′]Ap[3′], suppressing the expression of IFN-I through the cGAS-STING pathway (26, 27, 28). Crystal structures of VACV poxin and its complexes with 2′,3′-cGAMP and Gp[2′-5′]Ap[3′] elucidated how poxin recognizes and degrades 2′,3′-cGAMP (26). Although MPXV also contains a poxin, it is fused to a schlafen protein (26, 29). Whether the schlafen domain affects the function of the poxin domain and the exact molecular mechanisms of MPXV poxin-schlafen recognizing and degrading 2′,3′-cGAMP remain unknown. In this work, we investigated how MPXV poxin-schlafen and its poxin domain suppress the cGAS-STING signaling pathway and degrade 2′,3′-cGAMP. High-resolution crystal structures of MPXV poxin domain and its complex with Gp[2′-5′]Ap[3′] revealed the structural basis of 2′,3′-cGAMP degradation mediated by MPXV poxin-schlafen. We also investigated the substrate specificity of MPXV poxin-schlafen and elucidated why it degrades 2′,3′-cGAMP, but not other dinucleotides such as 3′,3′-cGAMP, c-di-AMP, and c-di-GMP. Furthermore, sequence alignment and structure comparison demonstrated that MPXV poxin-schlafen is a conserved target for the development of therapeutics against MPXV infection.

## Results

### MPXV poxin-schlafen degrades 2′,3′-cGAMP and suppresses cGAS-STING signaling

**Figure 1:**
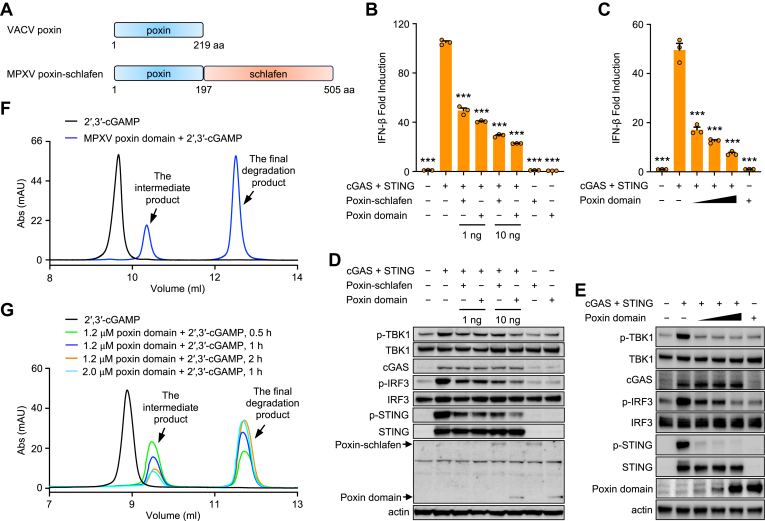
**MPXV poxin-schlafen suppresses cGAS-STING signaling *via* degrading 2′,3′-cGAMP.***A*, schematic representation of VACV poxin and MPXV poxin-schlafen. *B*, IFN-β luciferase reporter assays in HEK293T cells. The cells were transfected with 0.1 ng human cGAS, 1 ng human STING and the indicated MPXV poxin-schlafen or MPXV poxin domain plasmids, and luciferase signals were determined. *C*, IFN-β luciferase reporter assays in HEK293T cells transfected with 0.1 ng human cGAS, 1 ng human STING and 1 ng, 10 ng, and 30 ng MPXV poxin domain plasmids. *D*, western blot analyses of TBK1, STING, and IRF-3 phosphorylation in HEK293T cells transfected with cGAS, STING and the indicated MPXV poxin-schlafen or MPXV poxin domain plasmids, (*E*) western blot showing the phosphorylation of TBK1, STING, and IRF-3 in HEK293T cells transfected with cGAS, STING, and MPXV poxin domain plasmids. *F*, anion exchange chromatography analyses of 2′,3′-cGAMP degradation in the absence and presence of MPXV poxin domain. *G*, anion exchange chromatography analyses of 2′,3′-cGAMP degradation at different reaction time points and at different MPXV poxin domain concentrations. Data in *B* and *C* are mean ± s.e.m. and representative of three independent experiments. Each dot represents a technical replicate (n = 3). ∗∗∗*p* < 0.001, two-tailed Student’s *t* test. Abs, absorbance; AU, absorbance units; cGAS, cyclic GMP-AMP synthase; IFN-β, interferon-β; IRF-3, IFN regulatory factor-3; MPXV, monkeypox virus; STING, stimulator of interferon genes; TBK1, TANK-binding kinase 1; VACV, vaccinia virus.

Previous biochemical and structural studies demonstrated that VACV poxin specifically degraded the second messenger 2′,3′-cGAMP of the cGAS-STING innate immune pathway into Gp[2′-5′]Ap[3′] (26). To investigate whether MPXV poxin-schlafen also degrades 2′,3′-cGAMP and suppresses cGAS-STING signaling, we conducted IFN-β luciferase reporter assays in HEK293T cells in the presence or absence of poxin-schlafen (Fig. 1, *A* and *B*). The reporter assays showed that the IFN-β reporter was activated in cells transfected with cGAS and STING, but not in the control cells (Fig. 1*B*). On cotransfection with MPXV poxin-schlafen, the activation of IFN-β reporter was inhibited, indicating that MPXV poxin-schlafen suppresses cGAS-STING mediated induction of IFN-β. Notably, similar results were obtained after cotransfection with a plasmid expressing the poxin domain of MPXV poxin-schlafen (Fig. 1*B*). Moreover, MPXV poxin domain suppressed the reporter activation in a dose-dependent manner (Fig. 1, *B* and *C*). These results suggested that MPXV poxin domain was sufficient to inhibit the reporter activation and the schlafen domain did not affect the poxin domain in suppressing cGAS-STING mediated induction of IFN-β. Consistent with the results from the IFN-β reporter assays, immunoblot analyses showed that coexpression of MPXV poxin-schlafen or its poxin domain markedly reduced the phosphorylation of TBK1, STING, and IRF-3 that are all essential for the activation of the IFN-β reporter *via* the cGAS-STING pathway (Fig. 1, *D* and *E*).

To further demonstrate that the poxin domain of MPXV poxin-schlafen can degrade 2′,3′-cGAMP, we expressed and purified the poxin domain and established a cGAMP degradation assay *in vitro* (Fig. S1*A*). The reaction products were analyzed by anion exchange chromatography and mass spectrometry (MS) (Fig. 1, *F* and *G*). On incubation with the purified poxin domain, the UV absorption peak for 2′,3′-cGAMP disappeared and two new peaks were observed, indicating that 2′,3′-cGAMP was degraded and two new degradation products were generated (Fig. 1*F*). Meanwhile, the first peak gradually decreased and the second peak gradually increased with the extension of incubation time or the increase of poxin concentration, suggesting that the first peak was an intermediate product and the second was the final product (Fig. 1*G*). Indeed, the results of MS demonstrated that the first peak was the intermediate product Gp[2′-5′]Ap[2′-3′] and the second was the final product Gp[2′-5′]Ap[3′], which are consistent with previous studies showing that 2′,3′-cGAMP was first degraded into Gp[2′-5′]Ap[2′-3′] and then further degraded into Gp[2′-5′]Ap[3′] by VACV poxin (Table S1) (26). Collectively, these studies demonstrate that both MPXV poxin-schlafen and its poxin domain can suppress cGAS-STING signaling by degrading the second messenger 2′,3′-cGAMP.

### Crystal structure of MPXV poxin domain

High-resolution structure of MPXV poxin-schlafen can shed light on the degradation mechanism of 2′,3′-cGAMP. Therefore, we attempted to crystalize the poxin-schlafen fusion protein for structure determination. Unfortunately, no crystals were obtained, maybe due to the flexible linker between the poxin domain and the schlafen domain. The results of IFN-β luciferase reporter assays had demonstrated that the schlafen domain did not affect 2′,3′-cGAMP degradation mediated by the poxin domain. Therefore, we focused on the structure analyses of MPXV poxin domain to elucidate the mechanism of 2′,3′-cGAMP degradation and determined the crystal structure of MPXV poxin domain at 1.75-Å resolution (Table S2).

**Figure 2:**
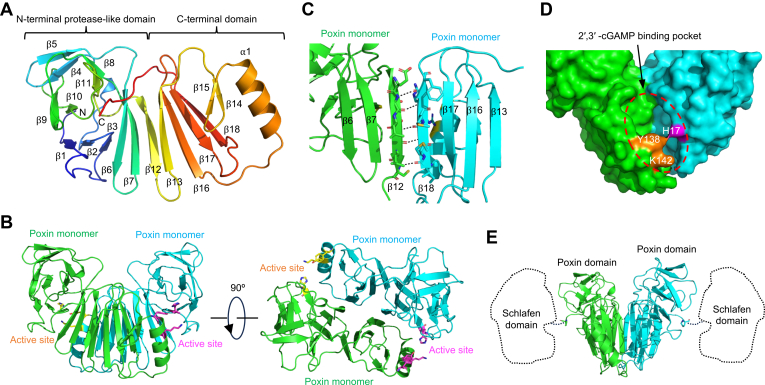
**Structure of MPXV poxin domain.***A*, ribbon representation of MPXV poxin domain. *B*, ribbon representations of MPXV poxin domain dimer. *Left*, side view into the active site of MPXV poxin domain dimer; *right*, *top* view of MPXV poxin domain dimer. Two MPXV poxin domain monomers are in *green* and *cyan*. The putative catalytic triad residues are shown by the *magenta* and *yellow* ball-and-stick models. *C*, the intermolecular β-sheet interactions between two monomers in the MPXV poxin domain dimer. The residues involved in forming the intermolecular β-sheet interactions are shown by ball-and-stick models. The *black dashed lines* indicate distances less than 3.5 Å. *D*, the pocket binding 2′,3′-cGAMP is surrounded by residues from one poxin monomer NPD (*cyan*) and another monomer CTD (*green*). The putative catalytic triad residues H17, Y138, and K142 are in *magenta* and *orange*, respectively. *E*, the structure model of MPXV poxin-schlafen. CTD, C-terminal subdomain; MPXV, monkeypox virus; NPD, N-terminal protease-like subdomain.

The structure shows that the poxin domain is composed of 18 β-strands and one α-helix (Fig. 2*A*). The first 12 β-strands constitute the N-terminal protease-like subdomain (NPD) while β13-β18 strands and α1 helix form the C-terminal subdomain (CTD) (Fig. 2*A*). Two MPXV poxin domains form a V-shaped homodimer (Fig. 2*B*). In the dimer, the β12 strand from one poxin monomer NPD forms antiparallel β-sheet interactions with the β18 strand from another monomer CTD (Fig. 2*C*). Therefore, these intermolecular interactions contribute to the formation of two continuous β-sheets, which stabilize the overall structure of the MPXV poxin dimer.

The pocket binding 2′,3′-cGAMP is surrounded by residues from one poxin monomer NPD and another monomer CTD (Fig. 2, *B* and *D*). Therefore, two monomers form two binding pockets, indicating that the poxin dimer can bind two 2′,3′-cGAMP simultaneously (Fig. 2, *B* and *D*). The putative catalytic triad (His17, Tyr138, and Lys142) is located at the edge of the binding pocket (Fig. 2*D*) (26). The C terminal tail of the poxin domain extends outward from the dimer and the last three residues are invisible in the structure, which suggest that the fused schlafen domains are likely located on the outside of the poxin dimer (Fig. 2*E*). Overall, MPXV poxin domain forms a dimer to recognize and degrade 2′,3′-cGAMP.

### The structural basis of 2′,3′-cGAMP degradation by MPXV poxin

**Figure 3:**
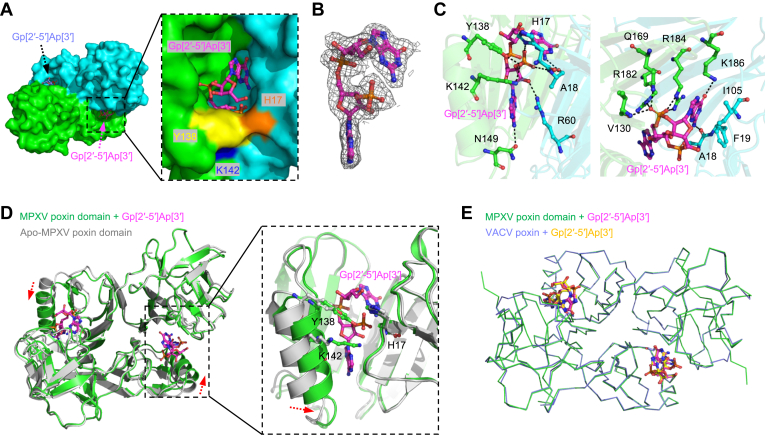
**Structure of MPXV poxin domain in complex with Gp[2′-5′]Ap[3′].***A*, surface representation of MPXV poxin domain dimer. Two MPXV poxin domain monomers are in *green* and *cyan*. Two Gp[2′-5′]Ap[3′] are shown by the *magenta* and slate ball-and-stick models. *B*, the density map of Gp[2′-5′]Ap[3′] contoured at 1 σ. Gp[2′-5′]Ap[3′] is shown by the *magenta* ball-and-stick model. *C*, interactions between Gp[2′-5′]Ap[3′] and MPXV poxin domain. Gp[2′-5′]Ap[3′] and residues are shown by ball-and-stick models. The *black dashed lines* indicate distances less than 3.5 Å. *D*, comparison of the structures of Apo-MPXV poxin domain (*gray*) and MPXV poxin domain-Gp[2′-5′]Ap[3′] complex (*green*). Gp[2′-5′]Ap[3′] is shown by the *magenta* ball-and-stick model. *E*, comparison of VACV poxin-Gp[2′-5′]Ap[3′] complex (PDB: 6EA9) and MPXV poxin domain-Gp[2′-5′]Ap[3′] complex structures. VACV poxin and MPXV poxin domain are shown by the *slate* and *green ribbons*. Gp[2′-5′]Ap[3′] are shown by the *magenta* and *yellow* ball-and-stick models. MPXV, monkeypox virus; PDB, Protein Data Bank; VACV, vaccinia virus.

To further investigate how MPXV poxin degrades 2′,3′-cGAMP, we determined the crystal structure of MPXV poxin domain in the presence of 2′,3′-cGAMP at 2.01-Å resolution (Table S2). The structure shows that the poxin domain forms a complex with the degradation product Gp[2′-5′]Ap[3′] instead of the substrate 2′,3′-cGAMP, which is consistent with the rapid degradation of 2′,3′-cGAMP by MPXV poxin domain in the *in vitro* assays (Figs. 1*F* and 3, *A* and *B*). Each substrate binding pocket of the poxin dimer binds one Gp[2′-5′]Ap[3′], forming a 2:2 complex (Fig. 3*A*). The adenine base of Gp[2′-5′]Ap[3′] inserts into the bottom of the binding pocket and interacts with the side chain of Asn149 from CTD *via* hydrogen bond and the guanidyl group of Arg60 from NPD *via* cation-π interaction (Fig. 3*C*). The 2′-OH of the adenine group forms hydrogen bond with the side chain of Arg60 (Fig. 3*C*). Meanwhile, the guanine base bends toward another pocket called guanine-binding pocket surrounded by Arg184 and Lys186 from CTD and Ala18, Phe19, and Ile105 from NPD (Fig. 3*C*). In this pocket, the guanine base forms hydrogen bonds with Ala18 and Lys186 and interacts with the guanidyl group of Arg184 *via* cation-π interaction (Fig. 3*C*). Furthermore, the 5′ phosphate group forms hydrogen bonds with the side chains of Gln169, Arg182, Arg184, and the main chain of Val130. The 3′ phosphate group interacts with the side chains of the putative catalytic triad His17, Lys142, Tyr138, and the main chain of Ala18 (Fig. 3*C*).

Compared to the apo-MPXV poxin domain structure, Gp[2′-5′]Ap[3′] binding induced notable conformational changes in the binding pocket (Fig. 3*D*). β14-β15 strands, α1 helix and several loops from the CTD moved toward the NPD of another poxin domain, which narrowed the binding pocket to clamp the substrate (Fig. 3*D*). After these structural rearrangements, two key residues Tyr138 and Lys142 in the putative catalytic triad approached the 3′-5′ phosphodiester bond for cleavage (Fig. 3*D*). The structures of VACV poxin and MPXV poxin domain in complex with Gp[2′-5′]Ap[3′] are very similar, suggesting that the mechanism of 2′,3′-cGAMP degradation by VACV poxin and MPXV poxin domain should be similar (Fig. 3*E*).

### Mutations of MPXV poxin affect 2′,3′-cGAMP degradation

**Figure 4:**
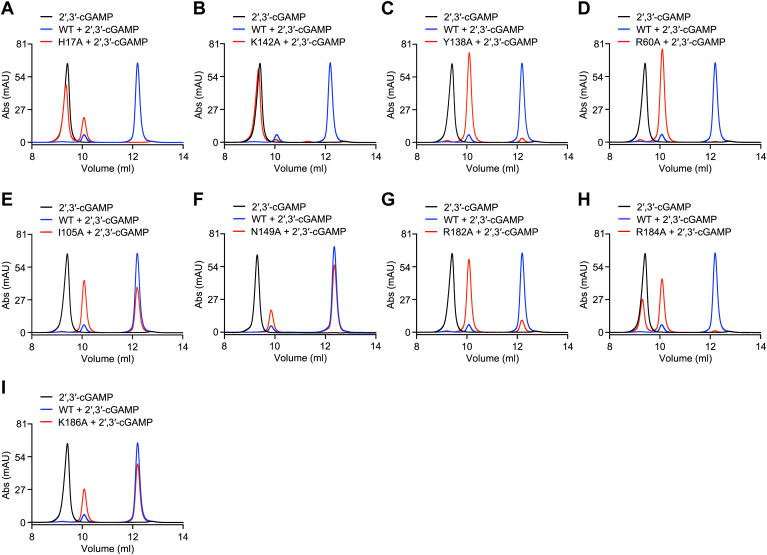
**Analyses of 2′,3′-cGAMP degradation by MPXV poxin domain mutants.***A–C*, anion exchange chromatography analyses of 2′,3′-cGAMP degradation by MPXV poxin domain mutants H17A, K142A, and Y138A. H17, K142, and Y138 forms the catalytic triad of MPXV. *D*–*I*, anion exchange chromatography analyses of 2′,3′-cGAMP degradation by MPXV poxin domain mutants R60A, I105A, N149A, R182A, R184A, and K186A. R60, I105, N149, R182, R184, and K186 are all involved in 2′,3′-cGAMP binding. Abs, absorbance; AU, absorbance units; MPXV, monkeypox virus.

Crystal structures of MPXV poxin domain and its complex with Gp[2′-5′]Ap[3′] reveal how the residues of the poxin domain bind and hydrolyze 2′,3′-cGAMP. To further confirm the results of structural and biochemical analyses, we mutated several key residues of MPXV poxin domain and purified these mutants for 2′,3′-cGAMP degradation studies (Fig. S1*B*). Mutating His17, a key residue of the putative catalytic triad to alanine dramatically reduced the degradation of 2′,3′-cGAMP to Gp[2′-5′]Ap[2′-3′], while mutating Lys142 to alanine nearly completely abolished 2′,3′-cGAMP degradation (Fig. 4, *A* and *B*). However, mutating Tyr138, another key residue of the putative catalytic triad to alanine nearly completely blocked the degradation of Gp[2′-5′]Ap[2′-3′] to Gp[2′-5′]Ap[3′] (Fig. 4*C*). These results indicate that His17 and Lys142 but not Tyr138 are key residues for the initiation of 2′,3′-cGAMP degradation by MPXV poxin.

Interestingly, mutating Arg60 that interacts with the adenine base to alanine blocked the degradation of Gp[2′-5′]Ap[2′-3′] to Gp[2′-5′]Ap[3′], suggesting that Arg60 is also a key residue for the second step of 2′,3′-cGAMP degradation (Fig. 4*D*). In MPXV poxin domain - Gp[2′-5′]Ap[3′] complex structure, the interaction between the guanidyl group of Arg60 and the 2′-OH of adenosine group may explain why Arg60 is critical for Gp[2′-5′]Ap[2′-3′] degradation to Gp[2′-5′]Ap[3′] (Fig. 3*C*). Furthermore, mutations I105A, N149A, R182A, R184A, and K186A all markedly reduced the degradation of 2′,3′-cGAMP (Fig. 4, *E–I*). Together, these activity assays further confirm that 2′,3′-cGAMP is degraded into the intermediate product Gp[2′-5′]Ap[2′-3′] and then further degraded into Gp[2′-5′]Ap[3′] by MPXV poxin.

### MPXV poxin degrades 2′,3′-cGAMP but not other cyclic dinucleotides

**Figure 5:**
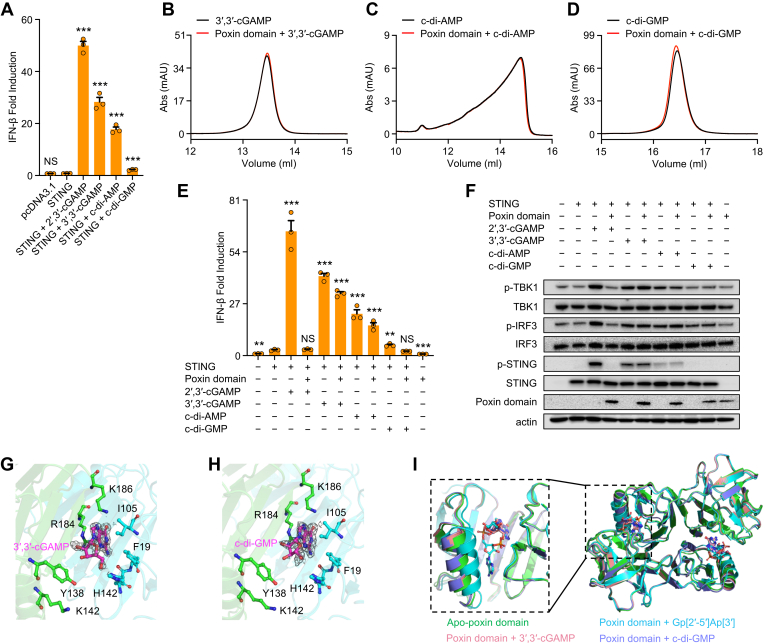
**MPXV poxin domain does not degrade 3′,3′-cGAMP, c-di-AMP, and c-di-GMP.***A*, IFN-β luciferase reporter assays in HEK293T cells transfected with human STING and stimulated with 2′,3′-cGAMP, 3′,3′-cGAMP, c-di-AMP, and c-di-GMP. *B*–*D*, anion exchange chromatography analyses of 3′,3′-cGAMP, c-di-AMP, and c-di-GMP degradation by MPXV poxin domain. *E*, IFN-β luciferase reporter assays in HEK293T cells transfected with human STING and/or MPXV poxin domain. The cells were stimulated with 2′,3′-cGAMP, 3′,3′-cGAMP, c-di-AMP, and c-di-GMP. *F*, western blot analyses of TBK1, STING, and IRF-3 phosphorylation in HEK293T cells transfected with human STING and/or MPXV poxin domain plasmids. The cells were stimulated with 2′,3′-cGAMP, 3′,3′-cGAMP, c-di-AMP, and c-di-GMP. *G* and *H*, the density maps of 3′,3′-cGAMP and c-di-GMP contoured at 1 σ. 3′,3′-cGAMP, c-di-GMP and residues are shown by ball-and-stick models. *I*, comparison of the structures of Apo-MPXV poxin domain (*green*), MPXV poxin domain-Gp[2′-5′]Ap[3′] complex (*cyan*), MPXV poxin domain-3′,3′-cGAMP complex (*pink*) and MPXV poxin domain-c-di-GMP complex (*slate*). Gp[2′-5′]Ap[3′], 3′,3′-cGAMP, and c-di-GMP are shown by ball-and-stick models. Data in *A* and *E* are mean ± s.e.m. and representative of three independent experiments. Each dot represents a technical replicate (n = 3). ∗*p* < 0.05, ∗∗*p* < 0.01, ∗∗∗*p* < 0.001, or not significant (*p* > 0.05), two-tailed Student’s *t* test. Abs, absorbance; AU, absorbance units; IFN-β, interferon-β; IRF-3, IFN regulatory factor-3; MPXV, monkeypox virus; STING, stimulator of interferon genes; TBK1, TANK-binding kinase 1.

In human cGAS-STING signaling pathway, STING is activated by the natural substrate 2′,3′-cGAMP synthesized by cGAS after sensing dsDNA(10). However, other cyclic dinucleotides produced by bacteria can also activate immune responses mediated by human STING (10, 30, 31, 32). Indeed, 3′,3′-cGAMP, c-di-AMP, and c-di-GMP also activated the IFN-β reporter in HEK293T cells expressing human STING (Fig. 5*A*). Notably, the reporter signals induced by 3′,3′-cGAMP and c-di-AMP were about half and one thirds of that induced by 2′,3′-cGAMP, respectively. Compared to 2′,3′-cGAMP, c-di-GMP only slightly activated the reporter (Fig. 5*A*). To test whether MPXV poxin domain also degrades these dinucleotides, we incubated the purified MPXV poxin domain with these dinucleotides and analyzed the reaction mixtures by anion-exchange chromatography (Fig. 5, *B–D*). The results showed that MPXV poxin domain did not degrade all these dinucleotides, indicating that MPXV poxin domain has a strict substrate specificity (Fig. 5, *B–D*). As expected, the IFN-β reporter activation induced by 2′,3′-cGAMP was completely abolished by coexpression of MPXV poxin domain. In contrast, MPXV poxin domain only resulted in slight reduction of the reporter signals induced by 3′,3′-cGAMP, c-di-AMP, and c-di-GMP (Fig. 5*E*). Consistent with the results from the IFN-β luciferase reporter assays, the phosphorylation of TBK1, STING, and IRF-3 induced by 2′,3′-cGAMP, not 3′,3′-cGAMP, c-di-AMP, and c-di-GMP was markedly reduced by coexpression of MPXV poxin domain in HEK293T cells (Fig. 5*F*).

After mixing MPXV poxin domain with these dinucleotides, we obtained crystals of MPXV poxin domain in complex with 3′,3′-cGAMP and c-di-GMP and solved the complex structures at resolutions of 1.81 Å and 1.94 Å, respectively (Table S2). In the structure of MPXV poxin domain in complex with 3′,3′-cGAMP, the guanine base is inserted into the guanine-binding pocket that can also bind the guanine base of 2′,3′-cGAMP (Fig. 5*G*). However, the phosphate groups and adenine base of 3′,3′-cGAMP are invisible, indicating that MPXV poxin domain binds 3′,3′-cGAMP and 2′,3′-cGAMP in distinct manners (Fig. 5*G*). Similar to MPXV poxin domain-3′,3′-cGAMP complex, only the guanine base in the guanine-binding pocket could be observed in the poxin domain-c-di-GMP complex (Fig. 5*H*). Although we also tried to crystalize MPXV poxin domain in the presence of c-di-AMP, no crystal was obtained. Compared to the apo- and Gp[2′-5′]Ap[3′]-bound MPXV poxin domain, the binding of 3′,3′-cGAMP and c-di-GMP does not induce conformational changes of the binding pocket that are critical for 2′,3′-cGAMP degradation, elucidating why MPXV poxin domain specifically degrades 2′,3′-cGAMP but not 3′,3′-cGAMP and c-di-GMP (Fig. 5*I*).

### MPXV poxin is a conserved target for inhibitor development

**Figure 6:**
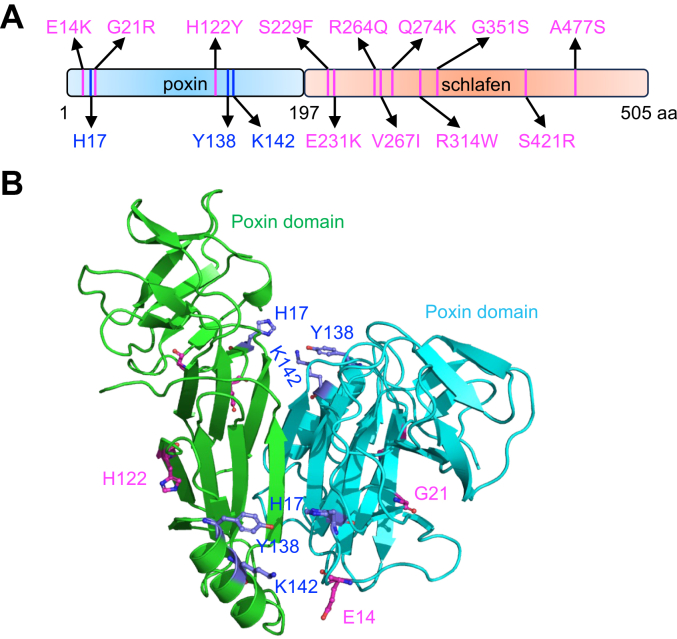
**Mutations of MPXV poxin-schlafen.***A*, schematic of MPXV poxin-schlafen showing the catalytic triad residues (*blue*) and all mutations (*purple*). *B*, locations of residues in MPXV poxin domain. The catalytic triad residues are shown by the slate ball-and-stick models. Residues that occur mutations in some MPXV poxin domain are shown by the *magenta* ball-and-stick models. MPXV, monkeypox virus.

To investigate whether mutations of MPXV poxin domain affect its activity for 2′,3′-cGAMP degradation, we conducted sequence alignment of over 60 sequences of MPXV poxin-schlafen from different countries or regions released by China National Center for Bioinformation (CNCB)/National Genomics Data Center (NGDC) (Table S3) (https://ngdc.cncb.ac.cn/gwh/poxvirus/release_genome)(33, 34, 35, 36). The results showed that no substitutions occurred in the catalytic triad (His17, Tyr138, and Lys142) and the residues (Arg60, Asn149, Arg182, Arg184, and Lys186) for 2′,3′-cGAMP binding (Fig. 6*A*). Only three substitutions (E14K, G21R, and H122Y) were found in some MPXV poxin domains (Fig. 6*A*). Mapping of these substitutions onto the structure of MPXV poxin domain showed that these three substitutions are located on the surface of the poxin domain and are not involved in 2′,3′-cGAMP degradation (Fig. 6*B*). Although nine substitutions were observed in the schlafen domain, they may not affect the function of the poxin domain because the wild-type schlafen domain does not affect 2′,3′-cGAMP degradation by the poxin domain (Fig. 6*A*). These results suggest that the conserved 2′,3′-cGAMP binding pocket of MPXV poxin can be targeted for drug development.

## Discussion

The outbreak of monkeypox caused by MPXV started in 2022 and continues to now (https://www.who.int/health-topics/mpox) (https://worldhealthorg.shinyapps.io/mpx_global/_w_02641a24967341c6aab56c7b40761811/#overview). However, how MPXV resists host immune surveillance facilitating persistent infection and effective transmission remains unclear. Previous studies showed that orthopoxviruses contain a nuclease called poxin that specifically hydrolyzes 2′,3′-cGAMP into Gp[2′-5′]Ap[3′]. As the second messenger of the cGAS-STING signaling pathway that can sense dsDNA virus infection, 2′,3′-cGAMP degradation by poxin suppresses the induction of IFN-I and facilitates orthopoxviruses blocking of innate immune responses (26, 27, 28). Here, we demonstrated that MPXV poxin-schlafen fusion protein and its poxin domain both could degrade 2′,3′-cGAMP and significantly reduced the activation of IFN-β reporter mediated by the cGAS-STING pathway in HEK293T cells. Consistent with our results, both the poxin from VACV and the poxin-schlafen from ECTV also could suppress cGAS-STING signaling and impair IFN-β reporter activation in HEK293T cells (26, 27). Deletion of poxin enhanced IRF-3 activation and IFN-I expression (37, 38). However, poxin or poxin-schlafen knockout had no effect on the replication of VACV or ECTV in cell culture, but significantly reduced VACV or ECTV replication in mice (26, 27). These results suggest that poxin likely blocks the spread of 2′,3′-cGAMP rather than inhibits interferon signaling in the primary infected cells (26, 27).

Surprisingly, two other studies found that poxin did not suppress the induction of IFN-I. One study showed that transfection of plasmid expressing VACV poxin did not inhibit the activation of IFN-β reporter mediated by the cGAS-STING pathway in HEK293T cells when they tried to screen genes for inhibition of the cGAS-STING pathway. Finally, they found that VACV E5 protein triggered ubiquitination of cGAS and proteasome-dependent degradation of cGAS, which inhibited IFN-I induction (24). In another study, they observed that MPXV poxin-schlafen protein did not inhibit IFN-β reporter activation, but interacted with STAT2 to suppress IFN-stimulated gene expression. Both the viral schlafen domain and the active site of the poxin domain were required for STAT2 sequestration (39). Now, we cannot explain why they obtained these different findings. Further studies of poxin are needed to resolve these questions.

Unlike VACV poxin, MPXV poxin is expressed as a fusion protein with a schlafen domain at the C terminal (26). Luciferase reporter assays showed that the schlafen domain had no effect on suppression of IFN-β reporter activation mediated by the poxin domain. Similar results were also observed from ECTV poxin-schlafen protein (27). The poxin domain, but not the schlafen domain was sufficient to suppress cGAS-STING signaling. However, the roles of the schlafen domain in orthopoxviruses remain unclear. Previous studies showed that the schlafen domain in orthopoxviruses is homologous to mammalian schlafen family members that are involved in cell differentiation and proliferation, antiviral immune responses, interferon-dependent gene expression and biological response, and so on (40, 41, 42, 43, 44, 45, 46, 47). These functions of mammalian schlafens may provide clues for further studies on the roles of the schlafen domain of orthopoxviruses.

The *in vitro* degradation assays demonstrated that MPXV poxin domain first degraded 2′,3′-cGAMP into an intermediate product and then the final product Gp[2′-5′]Ap[3′]. The intermediate product Gp[2′-5′]Ap[2′-3′] was further confirmed by MS. Similar results were also observed from VACV poxin degrading 2′,3′-cGAMP. Crystal structures of MPXV poxin domain and its complex with Gp[2′-5′]Ap[3′] reveal how MPXV poxin domain recognizes and degrades 2′,3′-cGAMP, but how Gp[2′-5′]Ap[2′-3′] binds MPXV poxin domain is still not clear. Mutating several key residues of MPXV poxin domain for 2′,3′-cGAMP degradation to alanines dramatically reduced or abolished Gp[2′-5′]Ap[2′-3′] degradation to Gp[2′-5′]Ap[3′]. Therefore, structural studies of these mutants with 2′,3′-cGAMP may reveal the structure of the intermediate of poxin hydrolysis.

The structures of VACV and MPXV poxins and their complexes with Gp[2′-5′]Ap[3′] are very similar, suggesting that the mechanism of 2′,3′-cGAMP degradation by poxin is conserved in orthopoxviruses. Therefore, the inhibitors targeting the active site of poxin should suppress the infection of all orthopoxviruses containing poxin or poxin-schlafen. Due to the critical roles of poxin in suppressing the immune responses mediated by the cGAS-STING pathway, the development of molecules that stimulate STING-mediated immune responses and resist degradation by poxin should be an effective strategy against orthopoxviruses infection. Therefore, many 2′,3′-cGAMP analogs such as fluorinated 2′,3′-cGAMP analogs MD1203 and MD1202D, dideoxy-2′,3′-cGAMP, dideoxy-2′,3′-cAAMP, and phosphorothioate substituted 2′,3′-cGAMP have been synthesized as STING agonists. Meanwhile, poxin could not degrade all these analogs (48, 49, 50). In this study, we observed that the second messengers such as 3′,3′-cGAMP, c-di-AMP, and c-di-GMP from bacteria also had the ability to activate the IFN-β reporter mediated by the cGAS-STING pathway and could not be degraded by MPXV poxin.

Crystal structures of MPXV poxin domain and its complexes with Gp[2′-5′]Ap[3′], 3′,3′-cGAMP and c-di-GMP elucidated why MPXV poxin domain can degrade 2′,3′-cGAMP but not 3′,3′-cGAMP and c-di-GMP. In structures of MPXV poxin domain in complex with 3′,3′-cGAMP and c-di-GMP, only the guanine bases of 3′,3′-cGAMP and c-di-GMP were observed in the guanine-binding pocket. The other parts of these two dinucleotides are invisible in the structures. Therefore, we speculate that the guanosine can be used as a lead compound to design and synthesize new inhibitors targeting poxin.

Due to sensing dsDNA in a sequence-independent manner, cGAS-STING innate immune pathway plays important roles in various physiological and pathological processes. When immune responses mediated by cGAS-STING pathway are not necessary, poxin can be used to degrade the second messenger 2′,3′-cGAMP and suppress the activation of the cGAS-STING pathway. Indeed, previous studies showed that the constitutive expression of P26, a poxin homolog from *Autographa californica* multiple nucleopolyhedrovirus (AcMNPV) prevented the production of IFN-β induced by baculovirus and enhanced baculovirus gene delivery by the attenuation of the antiviral activity. The incorporation of P26 into budded baculoviral vectors was a very promising tool to negatively modulate antiviral immune response and to improve the efficiency of baculoviral vectors for gene delivery in mammalian cells (51). In addition, poxin expression could inhibit cGAS-STING signaling in natural killer (NK) cells, thereby enhancing their cytotoxicity and antitumor activity (52).

In summary, these studies demonstrated that both MPXV poxin-schlafen and its poxin domain can degrade 2′,3′-cGAMP and inhibit cGAS-STING mediated IFN-β induction. The structural studies elucidated how MPXV poxin domain hydrolyzes 2′,3′-cGAMP but not 3′,3′-cGAMP, c-di-AMP, and c-di-GMP. These studies provide the structural basis for the development of poxin inhibitors to fight against MPXV infection.

## Experimental procedures

### Cell culture

HEK293T cells (American Type Culture Collection [ATCC] CRL-3216) were cultured in Dulbecco's modified Eagle's medium (1×) supplemented with 10% fetal bovine serum (VivaCell Biosciences), 100 U/ml penicillin, and 100 μg/ml streptomycin at 37 °C in a humidified atmosphere with 5% CO_2_. The cells tested negative for *mycoplasma* contamination.

### IFN-β luciferase reporter assays

The complementary DNAs (cDNAs) encoding full-length human cGAS and STING were cloned into a pcDNA3.1(−) vector. MPXV poxin-schlafen and poxin domain genes were cloned into a modified pcDNA3.1(−) vector with a C-terminal FLAG tag. All plasmids were verified by DNA sequencing. HEK293T cells were seeded in CoStar White 96-well plate (Corning) at 2.5 × 10^4^ cells per well and cultured at 37 °C with 5% CO_2_. After 24 h, the cells were transfected with IFN-β firefly luciferase reporter plasmid (20 ng per transfection), phRL-TK-Renilla luciferase plasmid (1 ng per transfection), pcDNA3.1-hcGAS plasmid (0.1 ng per transfection), pcDNA3.1-hSTING plasmid (1 ng per transfection), indicated amounts of pcDNA3.1-MPXV poxin-schlafen-FLAG plasmid or pcDNA3.1-MPXV poxin domain-FLAG plasmid using the transfection reagent Lipofectamine 2000 (Invitrogen) according to the manufacture’s manual. Empty pcDNA3.1(−) plasmid was used as transfection control and also added to normalize the amount of DNA in each transfection. After 16 h incubation, the cells were analyzed using the Dual-Glo Luciferase Reporter Assay Kit (Promega). Luminescence was quantified with the SpectraMax i3x microplate reader (Molecular Devices).

To investigate how the cyclic dinucleotides activate STING-mediated immune responses, HEK293T cells were transfected with IFN-β firefly luciferase reporter plasmid (20 ng per transfection), phRL-TK-Renilla luciferase plasmid (1 ng per transfection), pcDNA3.1-hSTING plasmid (1 ng per transfection) and/or pcDNA3.1-MPXV poxin domain-FLAG plasmid (10 ng per transfection). The cells were incubated for a further 12 h and then were treated with 30 ng/μl 2′,3′-cGAMP, 3′,3′-cGAMP, c-di-AMP, or c-di-GMP. Luminescence was quantified after 10 h stimulation.

All data were presented as mean ± s.e.m. A two-tailed Student’s *t* test assuming equal variants was used to compare two groups. The statistical significance between the indicated samples was designated as ∗*p* < 0.05, ∗∗*p* < 0.01, ∗∗∗*p* < 0.001, or not significant (*p* > 0.05).

### Western blot

HEK293T cells were seeded in 12-well plates, cultured at 37 °C for 24 h and then transfected with pcDNA3.1-hcGAS plasmid, pcDNA3.1-hSTING plasmid, indicated amounts of pcDNA3.1-MPXV poxin-schlafen-FLAG plasmid or pcDNA3.1-MPXV poxin domain-FLAG plasmid. After 16 h incubation, the cells were washed and resuspended with 1 × PBS. Then, the cells were collected by centrifugation at 2000*g* for 3 min and lysed in 200 mM Tris–HCl (pH 7.5), 150 mM NaCl, 1 mM EDTA, and 1% Nonidet P-40 supplemented with one complete EDTA-free protease inhibitor mixture tablet (Roche) and one PhosSTOP phosphatase inhibitor mixture tablet (Roche). After incubation on ice for 30 min and centrifugation at 21,130*g* for 10 min at 4 °C, the supernatants were collected for SDS-PAGE.

The proteins were separated by 4 to 20% SDS-PAGE gel and transferred to polyvinylidene fluoride membranes (Millipore). The membranes were blocked with 5% nonfat milk in 1 × PBS for 1 h at room temperature, followed by incubation with the primary antibodies that are as follows, anti-IRF-3 (Cell Signaling, Cat# 4302S, 1:1000), anti-IRF-3 phospho-Ser386 (Abcam, Cat# ab76493, 1:2500), anti-TBK1 (Cell Signaling, Cat# 3013S, 1:1000), anti-TBK1 phospho-Ser172 (Cell Signaling, Cat# 5483S, 1:1000), anti-STING (Cell Signaling, Cat# 13647S, 1:1000), anti-STING phospho-Ser366 (Cell Signaling, Cat# 19781S, 1:1000), anti-human cGAS (Cell Signaling, Cat# 15102S, 1:1000), anti-Flag (Cell Signaling, Cat# 14793S, 1:1000), anti-β-actin (Thermo Fisher Scientific, Cat# MA5-11869, 1:4000). After incubation with the primary antibodies overnight at 4 °C and washing with PBST (1×PBS and 0.1% Tween 20) three times, the membranes were incubated with the corresponding horseradish peroxidase (HRP)-conjugated secondary antibodies at 1:5000 dilution for 2 h at room temperature. Proteins of interest were visualized using a Tanon-4600 Automated Chemiluminescence Imaging System and the Western Lightening Plus ECL kit (PerkinElmer) according to the manufacturer’s protocol.

### Protein expression and purification

The cDNAs encoding MPXV poxin domain and mouse cGAS catalytic domain were cloned into a modified pET28(a) vector with an N-terminal His_6_-sumo tag. All proteins were expressed in *Escherichia coli* BL21 (DE3) cultured in Luria-Bertani (LB) broth supplemented with 50 μg/ml kanamycin at 37 °C. When the absorbance at 600 nm (A_600_) reached 1.0 to 1.2, the cells were induced with 0.4 mM isopropyl β-D-1-thiogalactopyranoside (IPTG) at 16 °C for 16 to 20 h. The cells were resuspended in the lysis buffer (50 mM Tris–HCl, pH 8.0, 300 mM NaCl). After sonication and centrifugation at 19,802*g*, 4 °C for 30 min, the supernatants were loaded onto a Ni^2+^-NTA Superflow column (QIAGEN). Then, the column was washed with the washing buffer containing 25 mM Tris-HCl, pH 7.5, 500 mM NaCl, and 25 mM imidazole and the target proteins were eluted with the elution buffer containing 25 mM Tris-HCl, pH 7.5, 150 mM NaCl, and 250 mM imidazole. The purified proteins were cleaved with sumo protease at 4 °C overnight. His_6_-sumo tag was removed using a Ni^2+^-NTA column. MPXV poxin domain and mcGAS catalytic domain in the flow through were further purified by gel-filtration chromatography using a HiLoad 16/600 Superdex 200 pg column (Cytiva). The purified proteins were concentrated to 15 mg/ml and stored at −80 °C.

All mutants of MPXV poxin domain were generated using a PCR-based technique with appropriate primers. The sequences of these mutants were confirmed by DNA sequencing. The mutants were expressed and purified as the WT protein.

### 2′,3′-cGAMP synthesis and purification

Subsequently, 10 μM purified mcGAS catalytic domain was incubated with 0.2 mg/ml salmon sperm DNA in the reaction buffer containing 20 mM Hepes pH 7.5, 150 mM NaCl, 5 mM ATP, 5 mM GTP, 5 mM MgCl_2,_ and 10 mM 2-mercaptoethanol (β-ME). The reaction lasted at least 24 h at 37 °C until ATP and GTP were exhausted. After removing cGAS and DNA by ultrafiltration and diluting with ddH_2_O, the reaction mixture was loaded onto a Q beads 6FF anion-exchange column (Smart-Lifesciences) pre-equilibrated with 50 mM NH_4_Ac. The column was washed with 50 mM NH_4_Ac and then 2′,3′-cGAMP was eluted with 300 mM NH_4_Ac. Finally, the eluted 2′,3′-cGAMP was lyophilized and stored at −20 °C.

### Poxin activity assays

All poxin activity assays were performed in the reaction buffer containing 50 mM Hepes pH 7.5, 150 mM NaCl, and 1 mM DTT. Briefly, 3 μM purified wild-type MPXV poxin domain or its mutants was incubated with 1 mM 2′,3′-cGAMP or other dinucleotides in the reaction buffer at 37 °C for 2 h. The reactions were stopped by boiling at 100 °C. After centrifugation at 21,130*g*, 4 °C for 5 min, the supernatants were analyzed on a Capto HiRes Q 5/50 anion-exchange column (Cytiva) preequilibrated with 50 mM Tris–HCl pH 8.5. The linear gradient of NaCl is 0 to 500 mM.

### LC-MS and LC-MS/MS analyses

LC-MS and liquid chromatography-tandem mass spectrometry (LC-MS/MS) experiments were performed on an Orbitrap Fusion mass spectrometer (Thermo Fisher Scientific Inc) coupled with a liquid chromatography system consisting an LC-40D XR quaternary liquid deliver pump, an SIL-40C XR auto-sampler, a CTO-40S column oven, and an SCL-40 communication interface (Shimadzu Co,). The column was an XSelect HSS T3 Column XP column (2.1 mm × 150 mm, 2.5 μm, Waters Co,). The mobile phases were 0.1% (v/v) formic acid aqueous solution as phase A and 0.1% (v/v) formic acid acetonitrile solution as phase B. The flow rate was 200 μl/min. The column was kept in the column oven at 45 °C. The gradient started at 100% phase A and 0% phase B. The composition was held for 2 min before phase B ramping to 100% in 40 min then kept for 5 min before decreased to 10% in 1 min.

The MS was operated in positive ion mode and the parameters for electrospray were as follows: voltage 3800 V, sheath gas 35, Aux gas 6, sweep gas 0, ion transfer tube temp 320 °C, and vaporizer temp 300 °C. The ion of dibutylphthalate (universally used plasticizer, m/z = 279.15909) was used as the internal m/z locker. MS spectra were acquired with a cycle time mode method with cycle time set to 2s. The m/z range for MS scan was 400 to 800 and resolution was 120,000. For MS/MS analyses, precursor ions were isolated (m/z isolation window = 2) and fragmented by higher-energy collisional dissociation (HCD) with a collision energy of 20%. The resolution of MS/MS scans was 30,000.0.

### Crystallization, data collection, and structure determination

All proteins were crystallized by sitting-drop vapor diffusion method at 4 °C. Crystals of MPXV poxin domain (15 mg/ml) were obtained by mixing the purified protein with equal volume reservoir buffer containing 0.1 M succinic acid pH 7.0, 15% w/v polyethylene glycol 3350. For the complexes, MPXV poxin domain was mixed with the dinucleotides at a 1:2 M ratio with a final concentration of MPXV poxin domain at ∼12 mg/ml. After mixing MPXV poxin domain with 2′,3′-cGAMP, the complex crystals were grown in 25% w/v polyethylene glycol 1500. Crystals of MPXV poxin domain in complex with 3′,3′-cGAMP were grown in 0.1 M Hepes pH 7.5, 12% w/v polyethylene glycol 3350. MPXV poxin domain in complex with c-di-GMP was crystallized in 0.1 M bicine pH 8.5, 8% w/v polyethylene glycol monomethyl ether 5000. The crystals were flash-frozen in liquid nitrogen in the reservoir solution containing 25% (vol/vol) glycerol or polyethylene glycol 400. Diffraction data were collected at Shanghai Synchrotron Radiation Facility (SSRF) beamline BL10U2 using a Eiger X 16M detector and were autoprocessed with the software XDS in BL10U2 (53). The structure of MPXV poxin domain was determined by molecular replacement using the structure of VACV poxin (Protein Data Bank [PDB]: 6EA6) as search model. The structures of MPXV poxin domain in complex with the dinucleotides were determined by molecular replacement using the solved MPXV poxin domain structure. All structures were manually adjusted using Coot and refined with Phenix package (54, 55). Details of data quality and structure refinement were summarized in Table S2. All structural figures were generated with PyMOL.

## Data availability

The atomic coordinate and structural factor of MPXV poxin domain have been deposited in the Protein Data Bank with the accession code 9XUJ. The atomic coordinates and structural factors of MPXV poxin domain in complex with Gp[2′-5′]Ap[3′], 3′,3′-cGAMP and c-di-GMP have been deposited in the Protein Data Bank (PDB) with accession codes 9XUP, 9XUT, and 9XUU respectively.

## Supporting information

This article contains supporting information.

## Conflict of interest

The authors declare that they have no conflicts of interest with the contents of this article.
