# Supplementary material for: Molecular mechanism of 2′,3′-cGAMP degradation by monkeypox virus poxin-schlafen protein
Source: J Biol Chem. 2026 May 15;302(7):113163. doi: 10.1016/j.jbc.2026.113163 (PMC13273817; doi:10.1016/j.jbc.2026.113163)
Supplement: Supplementary Material [file mmc1.docx]

**Molecular mechanism of 2′,3′-cGAMP degradation by monkeypox virus poxin-schlafen protein**

Yunxiao Huang^1^, Benzhen Duan^1^, Yang Xiao^1^, Xiaoman An^1^, Hongyu Zhao^1^, Jingwen Wang^1^, Fenglei Du^1^, Baoyu Zhao^1,*^

**1.** Shanghai Institute of Infectious Disease and Biosecurity, Key Laboratory of Medical Molecular Virology (MOE/NHC/CAMS), Shanghai Frontiers Science Center of Pathogenic Microorganisms and Infection, Department of Medical Microbiology and Parasitology, School of Basic Medical Sciences, Shanghai Medical College, Fudan University.

**Supplementary Figure 1**

**Supplementary Table 1**

**Supplementary Table 2**

**Supplementary Figure 1**


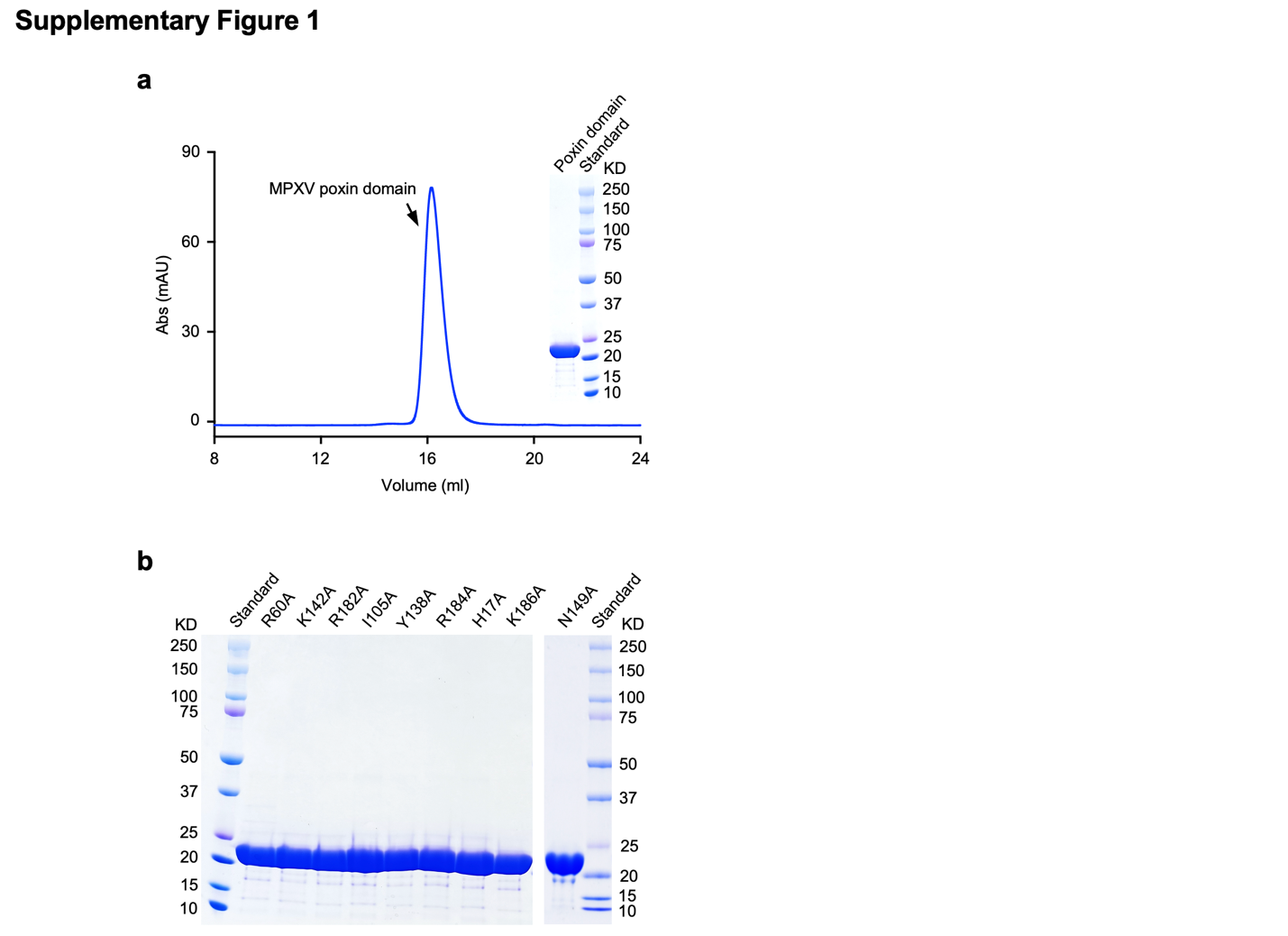


**Supplementary Figure 1** **| Analyses of MPXV poxin domain and its mutants**

(**a**) Gel-filtration chromatography and SDS-PAGE analyses of MPXV poxin domain. Abs, absorbance; AU, absorbance units.

(**b**)SDS-PAGE analyses of MPXV poxin domain mutants.

**Supplementary Table 1** Mass spectrometry analyses of the degradation products

| 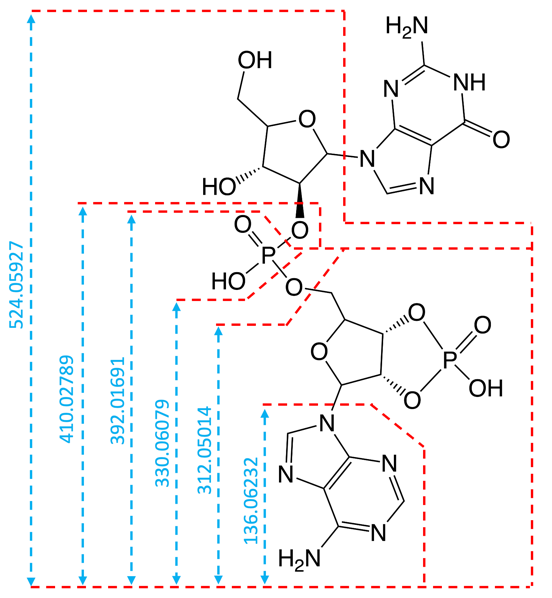  The intermediate product  (Molecular weight: 674.42) |   (LC-MS) |
| --- | --- |
|  |   (LC-MS/MS) |
| 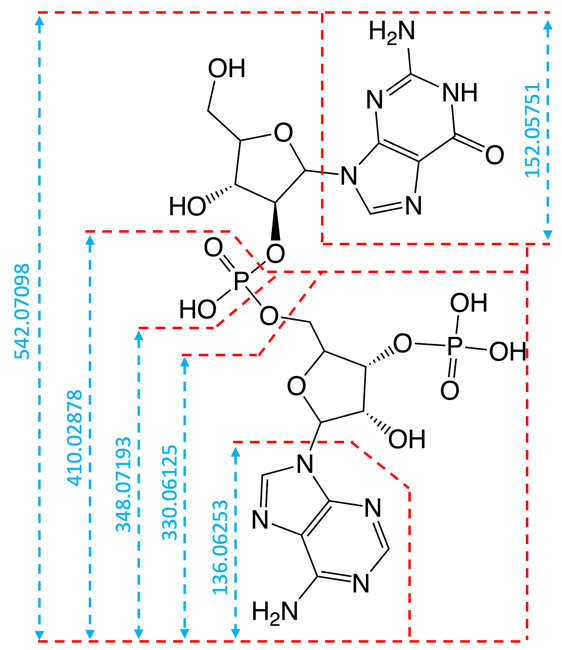  Gp[2'-5']Ap[3']  (Molecular weight: 692.43) |   (LC-MS) |
|  |   (LC-MS/MS) |

**Supplementary Table 2** Data collection and refinement statistics

|  | MPXV poxin domain | MPXV poxin domain  + Gp[2′-5′]Ap[3′] | MPXV poxin domain  + 3′,3′-cGAMP | MPXV poxin domain  + c-di-GMP |
| --- | --- | --- | --- | --- |
| **Data collection** |  |  |  |  |
| Space group | P2_1_ | P2_1_ | P2_1_ | P2_1_ |
| Cell dimensions |  |  |  |  |
| *a*, *b*, *c* (Å) | 54.24, 91.07, 94.11 | 61.98, 49.36, 75.32 | 54.18, 91.85, 93.66 | 54.29, 91.70, 93.66 |
|  *α* *β* *γ * (°) | 90.00, 90.08, 90.00 | 90.00, 90.82, 90.00 | 90.00, 90.32, 90.00 | 90.00, 90.45, 90.00 |
| Resolution (Å) | 1.75 (1.80 to 1.75) | 2.01 (2.06 to 2.01) | 1.81 (1.86 to 1.81) | 1.94 (1.99 to 1.94) |
| Unique reflections | 90,974 | 29,504 | 81,352 | 66,956 |
| *R*_merge_ | 5.0% (43.3%) | 23.0% (65.7%) | 13.9% (49.2%) | 6.8% (21.8%) |
| *R*_p.i.m._ | 2.2% (26.4%) | 11.3% (36.5%) | 6.2% (25.5%) | 3.0% (12.8%) |
| CC_1/2_ | 0.999 (0.828) | 0.975 (0.417) | 0.996 (0.557) | 0.998 (0.920) |
| *I* /σ*I* | 18.9 (2.6) | 9.6 (3.3) | 11.7 (3.7) | 17.5 (7.5) |
| Completeness (%) | 98.8 (91.5) | 97.9 (95.4) | 97.6 (97.0) | 98.6 (91.8) |
| Redundancy | 5.8 (3.5) | 5.3 (4.0) | 6.0 (4.6) | 5.7 (3.6) |
|  |  |  |  |  |
| **Refinement** |  |  |  |  |
| Space group | P2_1_ | P2_1_ | P2_1_ | P2_1_ |
| Resolution (Å) | 1.75 | 2.01 | 1.81 | 1.94 |
| No. Reflections used | 90,905 | 29,344 | 81,290 | 66,912 |
| *R*_work_/*R*_free_ (%) | 20.2/23.7 | 19.0/22.5 | 18.7/21.7 | 16.4/20.4 |
| No. atoms |  |  |  |  |
| Protein | 6,264 | 3,115 | 6,253 | 6,251 |
| Ligand/ion | 0 | 100 | 89 | 80 |
| Water | 743 | 259 | 884 | 832 |
| Average B-factors | 30.30 | 28.60 | 21.44 | 20.87 |
| R.m.s. deviations |  |  |  |  |
| Bond lengths (Å) | 0.006 | 0.007 | 0.007 | 0.007 |
| Bond angles (°) | 0.830 | 0.900 | 0.900 | 0.860 |
| Ramachandran plot, % residues in regions |  |  |  |  |
| Favored regions | 96.89 | 96.89 | 97.02 | 97.15 |
| Outliers | 0 | 0 | 0 | 0 |

**a.** One crystal was used to collect each of the dataset.

**b.** Values in parentheses are for the highest-resolution shell.
